# Iron–Quercetin Complex Preconditioning of Human Peripheral Blood Mononuclear Cells Accelerates Angiogenic and Fibroblast Migration: Implications for Wound Healing

**DOI:** 10.3390/ijms22168851

**Published:** 2021-08-17

**Authors:** Jiraporn Kantapan, Nampeung Anukul, Nipapan Leetrakool, Gwenaël Rolin, Jackie Vergote, Nathupakorn Dechsupa

**Affiliations:** 1 Molecular Imaging and Therapy Research Unit, Department of Radiologic Technology, Faculty of Associated Medical Sciences, Chiang Mai University, Chiang Mai 50200, Thailand; jiraporn.kan@cmu.ac.th; 2 Division of Transfusion Science, Department of Medical Technology, Faculty of Associated Medical Sciences, Chiang Mai University, Chiang Mai 50200, Thailand; nampeung.a@cmu.ac.th; 3 Blood Bank Section, Maharaj Nakorn Chiang Mai Hospital, Faculty of Medicine, Chiang Mai University, Chiang Mai 50200, Thailand; nleetrak@gmail.com; 4 Inserm Centre d’Investigation Clinique-1431 (Inserm CIC-1431), Centre Hospitalier Régional Universitaire de Besançon, F-25000 Besançon, France; grolin@chu-besancon.fr; 5 Inserm UMR1098, RIGHT Interactions Greffon-Hôte-Tumeur/Ingénierie Cellulaire et Génique, Etablissement Français du Sang en Bourgogne Franche-Comté, Université de Bourgogne Franche-Comté, F-25000 Besançon, France; 6 Laboratoire Signalisation et Transports Ioniques Membranaires (EA 7349), Faculté de Pharmacie, Université de Tours, F-37200 Tours, France; jackie.vergote@univ-tours.fr

**Keywords:** ex vivo expansion, endothelial progenitor cells, peripheral blood mononuclear cells, IronQ complex, stem cell tracking, MRI contrast agent

## Abstract

Cell-based therapy is a highly promising treatment paradigm in ischemic disease due to its ability to repair tissue when implanted into a damaged site. These therapeutic effects involve a strong paracrine component resulting from the high levels of bioactive molecules secreted in response to the local microenvironment. Therefore, the secreted therapeutic can be modulated by preconditioning the cells during in vitro culturing. Herein, we investigated the potential use of magnetic resonance imaging (MRI) probes, the “iron–quercetin complex” or IronQ, for preconditioning peripheral blood mononuclear cells (PBMCs) to expand proangiogenic cells and enhance their secreted therapeutic factors. PBMCs obtained from healthy donor blood were cultured in the presence of the iron–quercetin complex. Differentiated preconditioning PBMCs were characterized by immunostaining. An enzyme-linked immunosorbent assay was carried out to describe the secreted cytokines. In vitro migration and tubular formation using human umbilical vein endothelial cells (HUVECs) were completed to investigate the proangiogenic efficacy. IronQ significantly increased mononuclear progenitor cell proliferation and differentiation into spindle-shape-like cells, expressing both hematopoietic and stromal cell markers. The expansion increased the number of colony-forming units (CFU-Hill). The conditioned medium obtained from IronQ-treated PBMCs contained high levels of interleukin 8 (IL-8), IL-10, urokinase-type-plasminogen-activator (uPA), matrix metalloproteinases-9 (MMP-9), and tumor necrosis factor-alpha (TNF-α), as well as augmented migration and capillary network formation of HUVECs and fibroblast cells, in vitro. Our study demonstrated that the IronQ-preconditioning PBMC protocol could enhance the angiogenic and reparative potential of non-mobilized PBMCs. This protocol might be used as an adjunctive strategy to improve the efficacy of cell therapy when using PBMCs for ischemic diseases and chronic wounds. However, in vivo assessment is required for further validation.

## 1. Introduction

Cell-based therapy has recently become a focus of regenerative treatment for ischemic diseases and chronic wounds. Clinical improvements have been observed using autologous total mononuclear cells (MNCs) freshly isolated from bone marrow or peripheral blood. These clinical experiences have proven that cell-based therapy for vascular regenerations is safe, feasible, and effective [1]. However, the low efficacy of functional cells, due to the lower number and dysfunction found in patients, has limited its potential as a therapeutic tool [2]. In this study, we consider peripheral blood as a renewable cell source that can be retrieved from a readily accessible body compartment by a minimally invasive procedure. However, peripheral blood mononuclear cells (PBMCs) contain a very low percentage (<0.06%) of stem/progenitor cells [3,4]. Moreover, aging and disease also attenuate the number and functionality of these cells [5]. Therefore, the biggest challenge lies in enhanced cell expansion and cell therapeutic potential for successful and wider clinical applications using autologous cells, while controlling costs. 

Adult peripheral blood-derived stem/progenitor cells are of interest as a potential source of stem cells because they are known to transdifferentiate [6,7] and comprise the enriched fraction of endothelial progenitor cells (EPCs), which possess superior regenerative efficacy [8]. Moreover, peripheral blood-derived stem/progenitor cells can be obtained from autologous sources, without the need for painful bone marrow aspiration. Although some clinical trials have shown the potential benefits of peripheral blood-derived stem cells in patients with ischemic arterial disease [9,10,11,12], controversial issues regarding the clinical benefits of blood-derived stem cells have emerged. These might be due to the variety of cell isolation protocols and the type of cells administered to patients, such as either enriched progenitor fractions or whole mononuclear cell fractions.

Due to the heterogeneity in the peripheral blood mononuclear cell population, our focus here is on the critical functions of monocytes/macrophages. Monocytes are precursors of macrophages; monocyte-derived progenitor cells play a crucial role in angiogenesis and regenerative processes. Monocyte-derived cells with spindle shape characteristics are called fibrocytes, and endothelial progenitor cells have pro-angiogenic potential in vitro and in vivo. Fibrocytes contribute to tissue repair and angiogenesis [13]. They are characterized by the expression of both hematopoietic and stromal cell markers (collagen I, collagen III, cluster of differentiation 34 (CD34), and CD45). Peripheral blood monocytes serve as an enriched source of endothelial progenitor cells. The involvement of EPCs in angiogenesis is well known. EPCs secrete angiogenic factors related to differentiation into the endothelial cell [14]. These cells have been shown to affect vascular repair in ischemic hind limb models of vascular injury [15,16]. Moreover, monocyte cells can also transdifferentiate into endothelial [17], neuronal [18], and mature myeloid cells [19]. Many antigenic markers, including CD34, CD133, CD45, vascular endothelial growth factor receptor-2 (VEGF-R2), CD133, C-X-C Motif Chemokine Receptor 4 (CXCR4), CD14, and CD31, have been utilized to identify EPC populations [20]. Depending on microenvironment stimulations, macrophages can be polarized into diverse subtypes that play different roles (i.e., the pro-inflammatory M1 phenotype and anti-inflammatory M2 phenotype). The M2-subtype plays a central stimulatory role within angiogenesis and tissue repair. These M2-like macrophages secrete an angiogenic factor (e.g., vascular endothelial growth factor (VEGF), interleukin 10 (IL-10), IL-12, tumor necrosis factor-alpha (TNF-α), matrix metalloproteinases-9 (MMP-9), plasminogen activator inhibitor-1 (PAI-1), urokinase-type-plasminogen-activator (uPA), and monocyte chemoattractant protein-1 (MCP-1)) to regulate vessel organization and vascular surveillance, as evidenced by the irregular vessels in the absence of these macrophages [21,22,23,24,25,26].

Based on the rarity of therapeutic cells, to achieve clinically meaningful cell numbers and the superior quality of cultured cells, a practical approach based on peripheral blood progenitor cells, mandating their ex vivo expansion, is needed. Interestingly, dietary compounds which could promote angiogenic potential have increasingly been applied. Quercetin has been extensively studied due to its potential pharmacological properties and beneficial health effects [27]. In particular, quercetin has been reported to have cardioprotective qualities [28]. It is also used as a Chinese medicine for the treatment of heart disease [29]. Recent studies have reported that quercetin enhances the cell proliferation, osteogenic differentiation, and angiogenic factor secretion of healthy rat bone marrow mesenchymal stem cells (BMSCs) [29]. Moreover, quercetin has also been shown to modulate inflammation in humans through mechanisms involving macrophages, by enhancing the anti-inflammatory properties of M2 macrophages [30]. However, the poor water solubility, chemical instability, and low bioavailability of quercetin significantly limit its clinical applications [31]. The complexation of quercetin and a large number of metal ions has been reported. This indicates that its biological activities can be improved and increased, compared with free quercetin [32].

Recently, we introduced a novel bi-functional magnetic resonance imaging (MRI) probe, the so-called iron–quercetin complex (IronQ), as a new MRI contrast agent for tracking labeled cells. IronQ modulates the cellular characteristics of PBMCs to enrich the blood-derived spindle-shape-like cells (EPCs) during in vitro expansion. Simultaneously, there is the potential to visualize IronQ-labeled cells that have been transplanted with MR imaging via the paramagnetic properties of IronQ [33]. The iron–quercetin complex has some practical advantages over other types of MRI contrast agents when used for clinical purposes. (1) They can be easily prepared without the use of any toxic agents or expensive equipment. (2) They are non-toxic to cells, and can be enriched by the circulating progenitor cells in expanded culture. (3) They have the potential to be monitored by clinical magnetic resonance imaging. With the use of IronQ with the conventional culturing technique, blood-derived pro-angiogenic cells can be expanded, increasing their angiogenic potential, while tracking IronQ-labeled cells via MRI. To minimize the cost of the cell expansion method and the safety profile of the cell tracking procedure, IronQ was applied to PBMCs. The objective of this study was to examine the effect of the iron–quercetin complex and PBMC transformation on changes in surface markers and the cytokine and growth factors released. Furthermore, we evaluated the therapeutic potential of expanded cells, including their angiogenic and wound healing potentials. The IronQ-preconditioning technique may enhance the isolated unstimulated peripheral blood progenitor cells’ therapeutic potential for therapeutic vasculogenesis and tissue regeneration.

## 2. Results

### 2.1. PBMCs Cultured with the IronQ Complex Show the Accelerating Proliferation of Adherent Spindle-Shaped Cells and the Number of Early Outgrowth Colonies (CFU-Hill)

**Figure 1:**
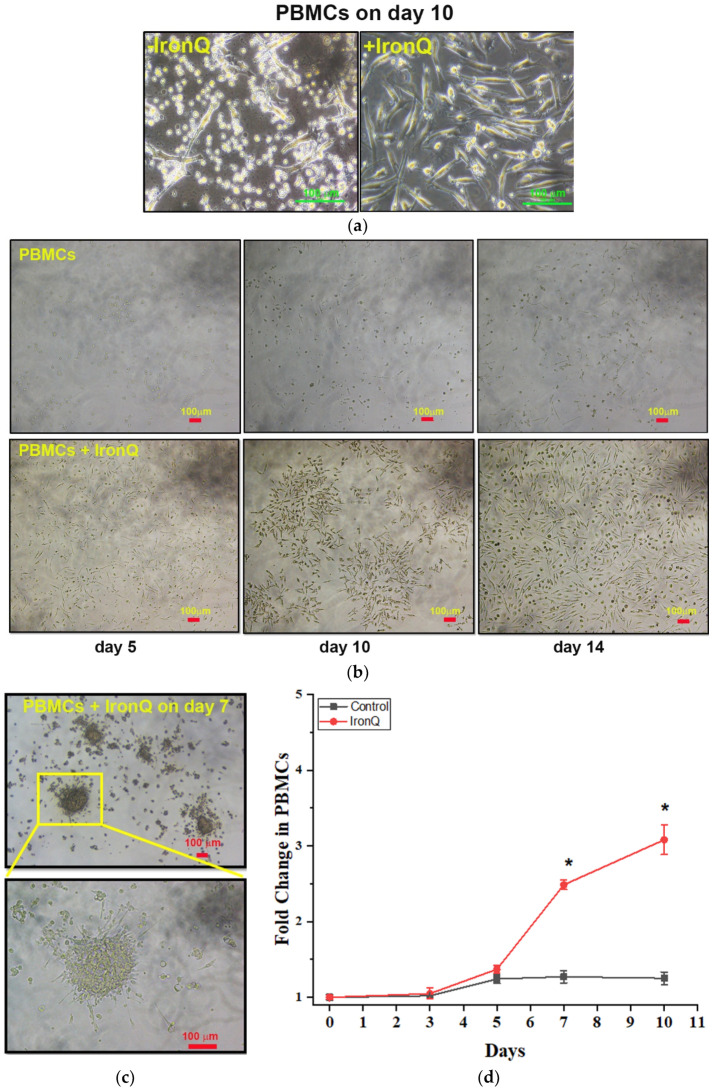
Proliferation and characteristics of the peripheral blood mononuclear cells (PBMCs) treated with the iron-quercetin complex (IronQ). (**a**) Representative phase-contrast images of the morphological states of PBMCs on day 10 of the culturing process. (**b**) PBMCs were cultured in the presence of 125 µg/mL of the IronQ complex. Morphological observation at different time points revealed that IronQ increased the number of adherent cells by almost 90% confluence on day 14. (**c**) PBMCs were cultured in the presence of 125 µg/mL of the IronQ complex. Colony-forming unit-Hill (CFU-Hill) or early outgrowth endothelial progenitor cells (EPCs) were observed after 7 days of culturing. The yellow box areas in the upper images (×40) were magnified in the lower images (×100). Scale bar = 100 µm. (**d**) A proliferation assay over 10 days of culturing revealed that the cells generated under IronQ conditions showed greater proliferation. Data are presented as the mean ± SD; *n* = 8. * *p* < 0.05.

Firstly, the appropriate concentration of the iron–quercetin complex (IronQ) was determined by cytotoxicity assay in a previous study [33]. We found that 125 µg/mL of IronQ complex was not toxic to peripheral blood mononuclear cells (PBMCs) when cultured for a long time (i.e., for one month). These concentrations were also used for cell tracking by MRI. Then, PBMCs were cultured in Roswell Park Memorial Institute (RPMI) 1640 medium with 10% FBS and 1% penicillin/streptomycin, without adding any specific growth factors, either in the absence (control) or in the presence of 125 µg/mL IronQ. Spindle shape cells appeared under both conditions, with different characteristics. Under the IronQ condition, the cells appeared as long spindle cells of considerable length (~100 µm). Inversely, the majority population of attaching cells in the untreated control group appeared to have a shorter spindle and be larger (Figure 1a). The total cell expansion of PBMCs cultured under the IronQ condition compared with the untreated control group was measured. The cell number in culture did not increase in the first 3 days of culturing, but increased slightly at day 5. The number of cells gradually increased from 1.3-fold on day 5 and up to 3-fold on day 14 after the cells were treated with IronQ, while in the untreated control group the number of cells reached a plateau phase (~1.3-fold) after day 5 of the culture period (Figure 1d). Morphological observations at different time points revealed that IronQ increased the number of adherent cells by almost 90% confluence on day 14 (Figure 1b), with an increasing number of early outgrowth colonies (CFU-Hill) at day 7 post-IronQ treatment (Figure 1c). CFU-Hill has been described by Hill et al., and is characterized by colonies displaying a central cluster of rounded and flat cells, with a radial arrangement of spindle-shaped cells (Figure 1c). These colonies were consistent with the endothelial progenitor cell (EPC) phenotype [34]. 

### 2.2. Cell Population Transition and Characterization of PBMCs Cultured under the IronQ Complex 

**Figure 2:**
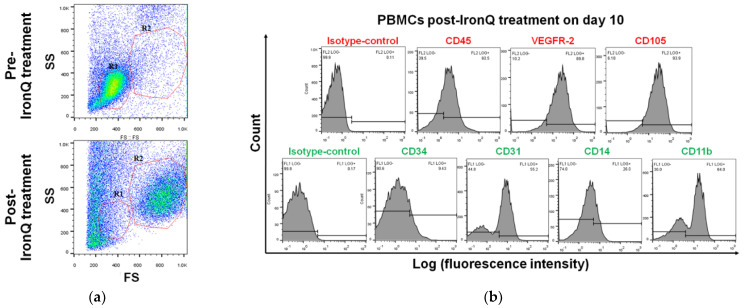

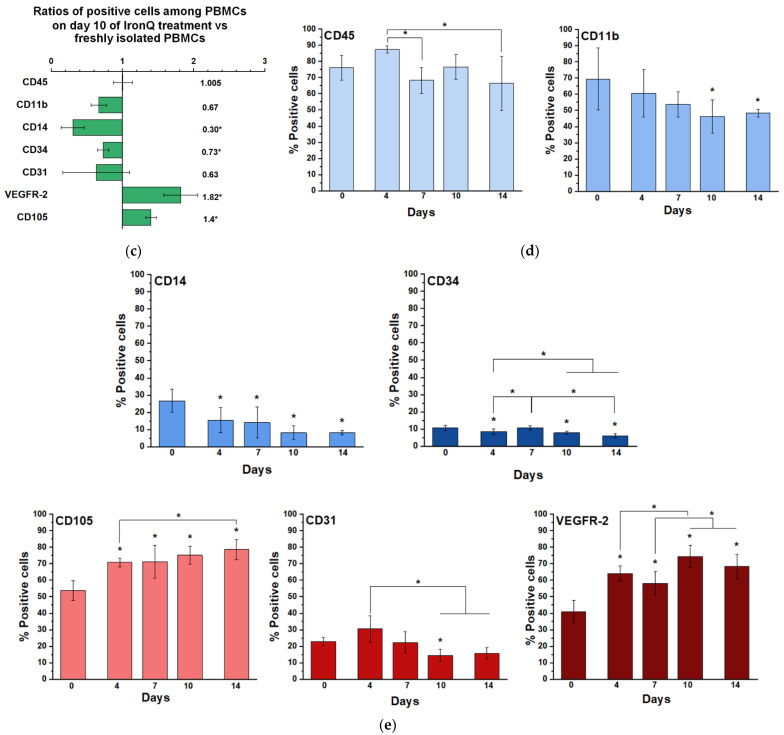
Flow cytometry analysis of pre-and-post IronQ PBMCs. (**a**) Scatter diagrams of pre-and-post IronQ PBMCs in flow cytometry. The red lines indicate the cellular-sized gates of lymphocytes and monocytes (R1), or the larger cells (R2). (**b**) Flow cytometry analysis for stem cells (cluster of differentiation 34 (CD34)), hematopoietic cells (CD14, CD11b, and CD45), and angiogenic (CD105, VEGFR-2, and CD31) markers in post-IronQ PBMCs (at day 10). (**c**) The bar graph shows the ratio of each percentage (%) of cell positivity in post-IronQ PBMCs (at day 10) to that of untreated PBMCs. The column represents the mean ± SD in each increase or decrease (*n* = 16), * *p* < 0.05. (**d,e**) Flow cytometric analysis of kinetic profiles of marker expression across population expansion. The data are presented as the mean ± SD (*n* = 8), * *p* < 0.05. PBMCs: peripheral blood mononuclear cells; IronQ: iron–quercetin complex; VEGFR-2: vascular endothelial growth factor receptor 2.

To further characterize PBMCs expanded under the IronQ complex condition, the surface expression of stem cell markers and markers related to angiogenesis was analyzed using flow cytometry. Based on the scatter diagram, PBMCs post-IronQ treatment (post-IronQ PBMCs) proportionally transitioned to a large cell population more commonly than in the PBMC untreated control group (pre-IronQ PBMCs) (Figure 2a). The red lines indicate the cellular-sized gates of lymphocytes and monocytes (R1), and the larger cells (R2). The proportion of each positive cell involved in the whole cells of the (R1) and (R2) gates was estimated. The percentage of cells expressing endothelial lineage cells was significantly increased in CD105 and VEGF receptor-2 (VEGFR-2) in the PBMC post-IronQ treatment group, whereas there was no significant difference between the two groups in the number of cells expressing CD31. The percentages of monocytes/macrophages (CD14 and CD11b) were decreased in the PBMCs post-IronQ treatment group versus the untreated control group. We observed a slight decrease in the stem cell marker CD34 in the PBMCs post-IronQ treatment group (Figure 2b,c). Altogether, the augmented frequency for VEGFR-2 or CD105 was considerably higher in the PBMC post-IronQ cells versus the monocytes/macrophages (CD14 and CD11b). These findings indicate that IronQ complex treatment promotes differentiation of circulating progenitor cells in peripheral blood into pro-angiogenic cells. We also evaluated the dynamic changes of seven different surface molecules during the culturing of PBMCs treated with the IronQ complex. The results are shown in Figure 2d. We found that the expression of the angiogenic markers CD105 and VEGFR-2 gradually increased, whereas the expression of CD31 markers remained expressed at variable levels throughout the culture period. Not surprisingly, the pan leukocyte marker CD45 stabilized with culture time, but the stem cell marker CD34 also followed this pattern. The monocyte/macrophage markers were diminished during the culturing. Interestingly, we observed that the marker expression reached its peak on day 10 of the culture period.

### 2.3. PBMCs Cultured with the IronQ Complex Secrete Vasculogenic, Anti-Inflammatory, and Wound-Healing Factors

**Figure 3:**
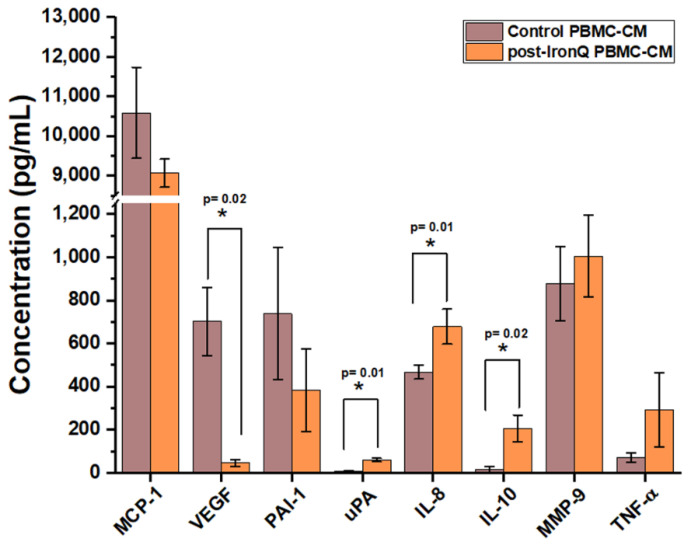
Treatment of PBMCs with the IronQ complex promotes the secretion of vasculogenic, anti-inflammatory, and wound-healing factors. Enzyme-linked immunosorbent assay (ELISA) measurement of cytokines in untreated PBMC-conditioned medium and IronQ-preconditioning PBMC-conditioned medium (at day 10). Data are expressed as means ± SD (*n* = 8). PBMCs: peripheral blood mononuclear cells.

Conditioned medium (CM) from the PBMCs post-IronQ treatment (post-IronQ PBMCs, at day 10) and untreated control PBMCs (control PBMC-CM) was evaluated for secreted angiogenic, anti-inflammatory, and wound-healing factors. Treatment with the IronQ complex stimulated secreted paracrine factors from the PBMCs. These molecules play a critical role in promoting angiogenesis and the wound-healing process. Analysis of the amounts of secreted factors was carried out by enzyme-linked immunosorbent assay (ELISA). The results (Figure 3) revealed the presence of pro-angiogenic factors, including IL-8 and urokinase plasminogen activator (uPA), which were significantly higher in the post-IronQ PBMC-CM than in the control PBMC-CM group, concurrently with the decrease in their inhibitor plasminogen activator inhibitor-1 (PAI-1). Inversely, the VEGF level was lower in the post-IronQ PBMC-CM than in the control PBMC-CM group. Additionally, the secretion of anti-inflammatory factor IL-10 was significantly higher in the post-IronQ PBMC-CM compared with the control PBMC-CM group. Treatment of PBMCs with the IronQ complex slightly increased the level of tumor necrosis factor-alpha (TNF-α) and matrix metalloproteinases 9 (MMP-9), but this change did not reach statistical significance, while the level of monocyte chemoattractant protein-1 (MCP-1) was essentially unchanged between the two groups.

### 2.4. PBMCs Cultured under the IronQ Complex and Their Conditioned Medium Show Strong Angiogenic Properties In Vitro

**Figure 4:**
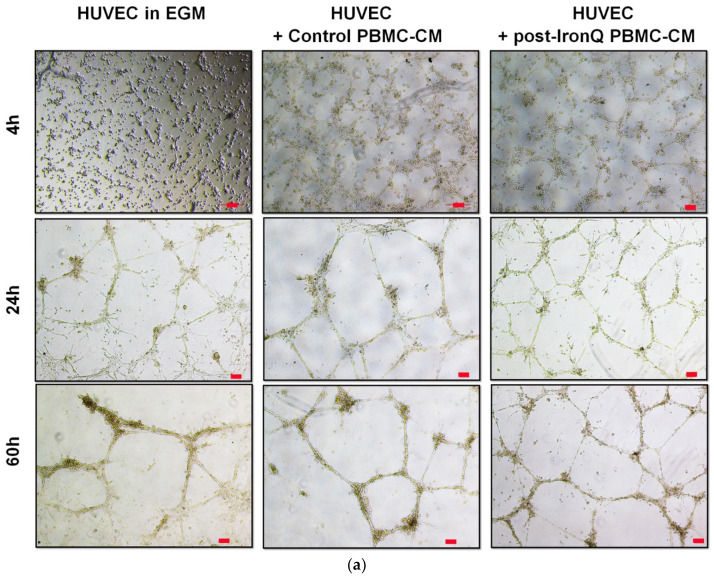

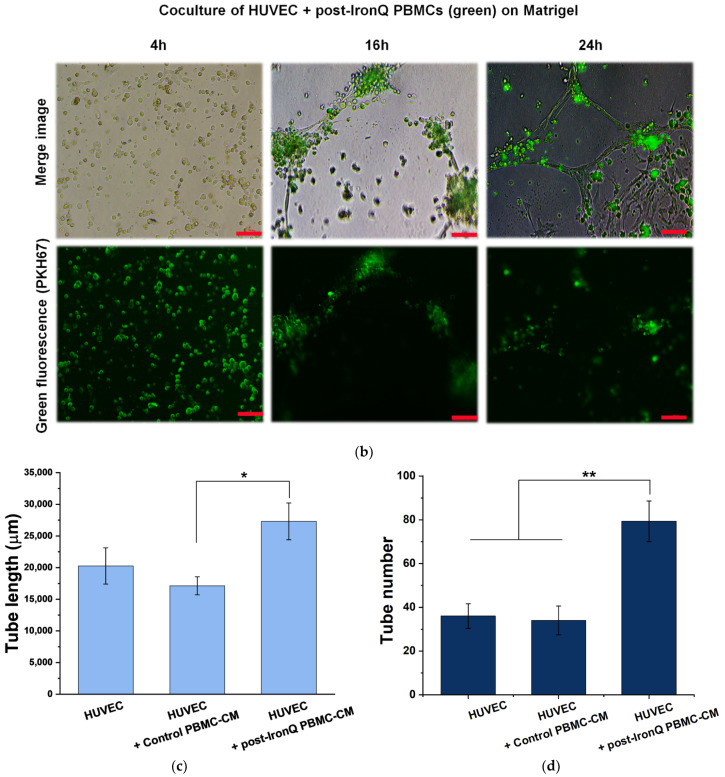
PBMCs treated with IronQ exhibit pro-angiogenic potential in the human umbilical vein endothelial cell (HUVEC) tubular network. (**a**) Tube formation ability of HUVECs cultured in conditioned medium from the PBMC control (control PBMC-CM), conditioned medium from IronQ-preconditioning PBMCs (post-IronQ PBMC-CM), or basal medium for endothelial cells (endothelial growth medium (EGM)). Scale bar = 100 µm, FOV (field of view). (**b**) Co-culture of HUVECs and PBMCs treated with IronQ (green fluorescence label) on Matrigel. Green fluorescence and merged images at 4, 16, and 24 h are shown. Scale bar = 100 µm. (**c**,**d**) Quantitative evaluation of tube length and tube number after the HUVECs were cultured in conditioned medium from PBMCs; the tube length and tube number were calculated in 4 random fields. Data are expressed as means ± SD. * *p* < 0.05, ** *p* < 0.01.

The pro-angiogenic potential of PBMCs treated with IronQ was investigated by in vitro angiogenic assay. Because treatment of PBMCs with the IronQ complex leads to secreted proangiogenic factors, we tested the effects of the conditioned medium of post-IronQ PBMCs on the promotion of tube formation in the Matrigel of human umbilical vein endothelial cells (HUVECs). HUVECs were seeded onto Matrigel in the presence of endothelial growth medium (EGM), conditioned medium from PBMCs (control PBMC-CM), or conditioned medium from IronQ-preconditioning PBMCs (post-IronQ PBMC-CM). HUVECs started to sprout and reorganize into tubular formations very early, within 4 h in the presence of conditioned medium of PBMCs, as compared to HUVECs cultured in EGM medium alone (Figure 4a). At 24 h of the time course, HUVECs in all conditions showed the formation of polygon structures as an endothelial tube network. The HUVECs appeared to lose their connections at 60 h in EGM medium, while the HUVECs cultured in post-IronQ PBMC-CM still showed well-reorganized tube formation (Figure 4a). We measured cumulative tube length at 24 h after seeding to estimate the stability of the tubular network. The quantitative results revealed a longer cumulative tube length in HUVECs cultured in post-IronQ PBMC-CM. No difference in the total tube length of HUVECs cultured in EGM medium or control PBMCs-CM was observed (Figure 4c). Moreover, HUVECs in post-IronQ PBMC-CM had a significantly increased number of tubules per field of view than those in EGM medium or control PBMC-CM (Figure 4d). These findings suggested that the treatment of PBMCs with IronQ led to secretion of proangiogenic agents that support the tubular formation of endothelial cells. 

We assessed the angiogenic potential of the PBMCs treated with IronQ in vitro. These PBMCs were labeled with green fluorescence (PKH-67), and then co-cultured with HUVECs on Matrigel. IronQ-preconditioning PBMCs colocalized and firmly attached to the endothelial tube network. They also participated at the junction point of the endothelial tubular network (Figure 4b). These results revealed that, when co-cultured, IronQ-preconditioning PBMCs secreted angiogenic factors that preferentially augmented the tubular network, and these post-IronQ PBMCs were able to stabilize the networks. However, these expanded PBMCs did not form a network on their own.

### 2.5. Conditioned Medium from PBMCs Cultured under the IronQ Complex (Post-IronQ PBMC-CM) Promotes Migration of Human Umbilical Cord Vein Endothelial Cells 

**Figure 5:**
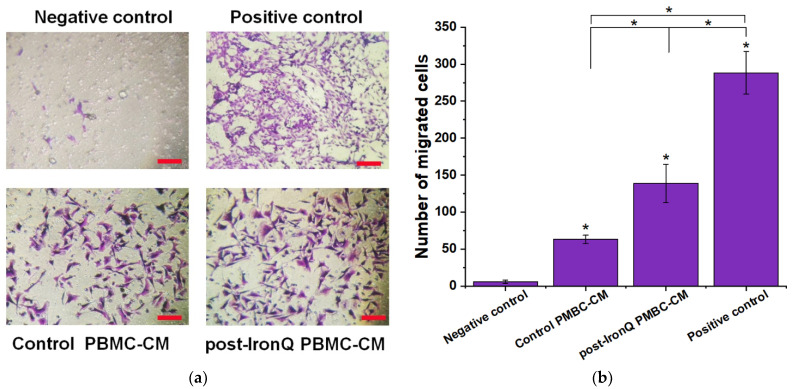
The effect of IronQ-preconditioning PBMC secreted factors on the migration of HUVECs. The HUVECs were cultured in the upper chamber of the Transwell chambers, with serum-free medium (0.1% FBS), and the lower chambers were filled with 0.1% FBS medium (negative control), 0.1% FBS + control PBMC-CM, 0.1% FBS + post-IronQ PBMC-CM, or 10% FBS medium (positive control). (**a**) After 24 h, the number of cells on the lower surface of the upper chamber was counted under an inverted microscope at ×100 magnification. (**b**) Quantification of HUVEC cell migration (violet stained cells) using a Transwell chamber. The quantitative evaluation of the number of cells migrated to the lower surface of the Transwell chamber under different conditions after 24 h of the culture period. Data are expressed as means ± SD (*n* = 8). * *p* < 0.05.

Since tube formation involves the migration of HUVECs, we tested the chemotactic response of HUVECs to post-IronQ PBMCs secreted factors using a Transwell migration assay. After 24 h, HUVEC migration in response to angiogenic factors secreted by IronQ-preconditioning PBMCs (post-IronQ PBMC-CM) was enhanced 3 to 30-fold (*p* < 0.05) over migration in response to the conditioned medium from control PBMCs (control PBMC-CM) or the negative control of 0.1% FBS medium alone. The numbers of migrating HUVECs in the control PBMC-CM were significantly greater than those in the negative control group. Not surprisingly, the highest number of migrating cells were observed for the positive control group (10% FBS medium), which had the greatest promoting effect (Figure 5a,b).

### 2.6. Conditioned Medium from PBMCs Cultured under the IronQ Complex (Post-IronQ PBMC-CM) Induces the Migratory Ability of Fibroblast Cells

**Figure 6:**
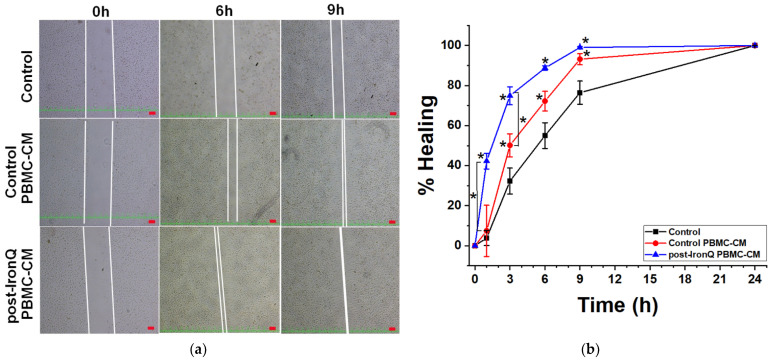
Effects of the conditioned medium of IronQ-preconditioning PBMCs on fibroblast migration in a wound scratch assay. (**a**) Representative images of scratch assays of L929 fibroblasts immediately after the scratches had been made (0 h) and then after 6 and 9 h in the presence of PBMC-CM, versus ordinary medium. Scale bar = 100 µm. (**b**) Scratch closure was measured as the width of each scratch using Image J software. Each time point was normalized to the image perimeter at 0 h, and reported as a percentage of healing. The results are expressed as means ± SD (*n* = 8). * *p* < 0.05.

To investigate whether the PBMC secreted factors affect the capacity of cell migration on fibroblasts, a wound closure migration assay was carried out. L929 fibroblast cells were grown to confluency, then scratched and treated with ordinary medium or PBMC secreted factors from conditioned medium over the time course of 24 h. As shown in Figure 6, the post-IronQ PBMC-CM induced a significantly shorter timeframe of L929 fibroblast migration, as compared to control PBMC-CM or ordinary medium, at the time points of 1 and 3 h (Figure 6b). After 9 h, scratched wound closure was almost completely fulfilled in the L929 fibroblast cultures exposed to both conditioned mediums (post-IronQ PBMC-CM and control PBMC-CM), as compared to the ordinary medium, which showed about 75% closure, before 100% closure after 24 h (Figure 6a,b). This result indicated that post-IronQ PBMC-CM contained factors that promoted fibroblast cell migration.

## 3. Discussion

At present, cell-based therapy is generating significant interest around the repair of ischemic damage. However, its clinical application remains limited due to the rarity of regenerative cells. Another challenge facing cell-based therapy is identifying the outcome and effectiveness, and real-time in vivo monitoring, of the transplanted cells [35]. Fortunately, the development of molecular magnetic resonance imaging (MRI) technology provides new approaches for the high-sensitivity study of transplanted cells’ therapeutic effects by non-invasive dynamic monitoring [36]. Our previous studies investigated the novel MRI contrast agent synthesis and characterization of the iron–quercetin complex, or IronQ. IronQ is a positive contrast for a T1-weighted MR image. We are also currently undertaking research on the safety and biodistribution of IronQ in an animal model. The prominent characteristic of IronQ is the high efficiency of loading into the cells, and the magnetically labeled mononuclear cells were visualized by a clinical 1.5 T MR scanner when the cell quantity was more than 2000 cells/µL. Interestingly, the effectiveness of visualizing the IronQ labeled cells was still sufficient when the labeled cells were in the culture for 21 days. It has been shown that IronQ can act as a stimulating agent by favoring the proangiogenic cell differentiation of PBMCs. Moreover, IronQ is highly sensitive and has no toxicity, but enhances the therapeutic efficiency of labeled cells [33]. These results suggest that IronQ is the most outstanding theranostic agent in applications of cell-based therapy. Recently, theranostic agents, including MRI contrast agents, have been developed for the purpose of diagnosis and therapy at the same time [37]. For example, a tumor specific magnetic nanoparticle-loaded doxorubicin agent has been developed by various research groups in the imaging and treatment of cancer [38,39]. 

In the present study, we have demonstrated that IronQ preconditioning enhances the in vitro functions of human peripheral blood mononuclear cells (PBMCs). We found that cultures of PBMCs under IronQ conditions were enriched with proangiogenic cells and blood-derived cells with regenerative capacity. Preconditioning PBMCs with IronQ enhanced the therapeutic potential of PBMCs, as they secreted important cytokines and growth factors supporting revascularization and tissue repair. The use of IronQ preconditioning produced an increase of a spindle-shaped cell type with a surface marker profile characterized by proangiogenic cells, comprising, for example, CD31+, CD105+, and VEGFR-2+ cells. Freshly isolated PBMCs cultured in an endothelial growth factor condition for a short culture period of 4–7 days exhibited a spindle cell-like morphology with a mixed expression profile of CD31, VEGFR-2, CD105, and von Willebrand factor—the so-called “early outgrowth endothelial progenitor cells” (early EPCs), or proangiogenic cells (PACs). Early EPCs contribute to angiogenesis mainly via paracrine signaling mechanisms. Early EPCs secrete multiple proangiogenic cytokines and growth factors augmenting endogenous vessel growth, but they fail to differentiate into endothelial cells [40]. A recent study showed that transplantation of peripheral blood-derived early EPCs was positive for endothelial markers such as CD31, VEGFR-2, von Willebrand factor, and CD105 in patients with acute myocardial infarction. Clinical improvements have been observed using these therapeutic cells, including decreased infarct size and an increase in the ejection fraction [1,41]. Culturing PBMCs under IronQ conditions also resulted in enhanced proliferation and PBMC population transition. The results demonstrated that PBMCs cultured with IronQ doubled their population number after 7 days of culturing, with a continued increase until day 10, while the number of PBMCs in the control group progressively decreased with increased time in culture (Figure 1 and Figure 2). The complex process of tissue repair is multistep, including angiogenesis and tissue regeneration, and is tightly controlled by the crosstalk between the cell populations in such a system [42]. Therefore, using a single cell type may be insufficient for successful treatment. There is growing evidence that angiogenesis is a critical process in tissue repair and regeneration. Dashtimoghadam et al. demonstrated that multifunctional cell therapy microcarriers containing mesenchymal stem cells (MSCs), endothelial cells, and vascular endothelial growth factor (VEGF) enhance the regeneration of bone tissue in vivo [43]. New blood vessel formation is a prominent process during tissue repair. It has been reported that the combined use of stem cells and endothelial cells more effectively harnesses the therapeutic effect for cardiac damage regeneration as compared to using stem cells or endothelial cells alone [44]. This suggests that the pro-angiogenesis condition is a critical factor for promoting tissue regeneration.

Our study adopted a heterogeneous cell mixture model of mononuclear cells to allow for crosstalk between all components of PBMCs. We hypothesized that IronQ would stimulate the mononuclear cells to amplify cytokine signals and crosstalk between the significant cell populations in such a system. The cytokines secreted from PBMC treatment with IronQ are well known for their capacity to promote angiogenesis, and include IL-8 and uPA. These angiogenic factors are potently secreted from various monocyte-derived angiogenic cells, including EPCs, pericytes, monocytes, and macrophages [13,45,46]. Additionally, IronQ can stimulate mononuclear cells to produce cytokines that contribute to regulating T cell differentiation, such as IL-10. This cytokine is produced by M2 macrophages and induces Th2 and Treg lymphocyte functions [47]. M2 macrophages ameliorate anti-inflammatory and immune-suppressive phenotypes, and they promote angiogenesis and tissue repair. It has been reported that intracellular iron status acts in modulation of macrophage plasticity and polarization. A recent study revealed the influence of iron on innate immune and macrophage polarization, in vivo and in vitro. The researchers found that an iron-rich status promotes M2 subtype macrophages and impairs the M1 subtype activated response to LPS-induced pro-inflammation [48]. Therefore, IronQ, containing iron, might stimulate and load intracellular mononuclear cells, resulting in PBMCs transforming into alternative M2 macrophage cells. A result of cytokines secreted by M2 macrophages (IL-10) is the regulation of T lymphocyte transformation into phenotypically polarized regenerative subsets (Th2 and Treg lymphocytes). Interestingly, IronQ treatment can induce PBMCs to expand proangiogenic cells and change blood cells into the cells responsible for vascular and tissue regeneration. From the IronQ treatment results of various combinations of therapeutic cells and secreted cytokines, we can conclude that cytokines play a synergistic role in the priming process. However, future experiments may involve closer examination of responsible factors and cells in IronQ treatment for the generation of therapeutic cells.

Growing evidence from recent studies strongly highlights the paracrine effect of the transplanted cells in cell therapy [49]. In our research, IronQ preconditioning of PBMCs promoted angiogenesis and tissue repair, mainly in a paracrine manner. The conditioned medium analysis obtained from post-IronQ treated PBMCs contained a remarkable amount of crucial proangiogenic cytokines, such as IL-8 and uPA. Importantly, IL-8 is a well-known proangiogenic cytokine and plays a significant role in angiogenesis. Regarding its association with angiogenesis, the therapeutic targeting of IL-8 is currently under research for cancer therapy [50]. The angiogenic property of IL-8 is involved in endothelial cell proliferation and capillary tube organization [51]. Additionally, urokinase plasminogen activator (uPA), which increased more than 10-fold in post-IronQ PBMC conditioned medium, plays a role in angiogenesis through proteolytic degradation of the extracellular matrix, which facilities the subsequent proliferation and migration of endothelial cells [52]. This finding indicated that post-IronQ treated PBMCs secreted proangiogenic factors. This may have been reflected in our observation of enhanced tube formation and migration of HUVECs after treatment with conditioned medium from post-IronQ treated PBMCs. However, VEGF, one of the main proangiogenic growth factors, was not elevated but reduced in post-IronQ treated PBMCs, relative to control PBMCs. A negative feedback mechanism might explain the reduction of the VEGF level, through a highly secreted TNF-alpha in post-IronQ treated PBMC conditioned medium. Apart from angiogenesis, uPA also plays a vital role in the wound-healing process, through aiding fibrin dissolution and promoting the migration, proliferation, and adhesion of various critical cells to the wound site during the early phases of wound healing [53]. The binding of uPA to the receptor in the target cell surface (uPAR) can initiate vitronectin- or integrin-mediated cell adhesion and migration, and eventually angiogenesis and tissue repair [54]. In vitro studies have shown that uPA promotes cell migration towards the wounded region [55]. Herein, IronQ-treated PBMCs secreted a higher level of uPA (*p* = 0.01). This phenomenon may be reflected in the findings of enhanced migration of L929 fibroblasts after culturing in post-IronQ treated PBMC conditioned medium (Figure 6).

Most notably, in our findings the secreted level of TNF-α (*p* = 0.74) and MMP-9 (*p* = 0.20) did not reach statistical significance in post-IronQ treated PBMCs compared to control PBMCs. The possible reason for this might be due to the variation in secretion levels between the subjects, as the cytokine was measured in individual subjects. Tumor necrosis factor-alpha (TNF-α) is a secretory product of activated macrophages, and a crucial proinflammatory mediator. However, TNF-α has been implicated in angiogenesis during inflammation, wound repair, and tumor growth [56]. It has been reported that TNF-α mediates crosstalk between macrophages and ECs at sites of inflammation, and where enhanced and temporally regulated angiogenic sprouting begins [57]. TNF-α may support angiogenesis via induced expression of proangiogenic genes, such as VEGFR-2, while blocking signaling through VEGFR-2. This results in delayed angiogenesis in the acute phase of the inflammatory response. Therefore, TNF-α can exert pro- or anti-angiogenic effects depending on its expression during the angiogenic process [57]. Furthermore, TNF-α is also a potent fibroblast chemoattractant and stimulates fibroblast proliferation, and the local application of TNF-α in collagen-based biomaterials can promote the healing of injured tissue [57,58,59].

The recruitment and activation of monocytes/macrophages within the ischemic tissues is essential for the tissue repair process [60]. MCP-1 is associated with monocyte recruitment, and has been considered a crucial proangiogenic factor [61]. Furthermore, these secretory cytokines are also known to be important in wound healing [62,63]. Following ischemic damage, the most frequently observed chemokine is monocyte chemotactic protein-1 (MCP-1). A recent study in the hindlimb ischemic model has shown the benefit of administering exogenous MCP-1 to increase blood flow to the ischemic tissue via increased monocyte/macrophage recruitment and augmentation of the development of collateral neovascularization [64]. Furthermore, MCP-1 has been shown to mobilize and transdifferentiate mononuclear monocyte lineage cells into endothelial-like cells [65]. These results suggest that PBMC treatment with the IronQ complex promotes PBMCs to secrete high levels of growth factors and cytokines that are important for angiogenesis and the wound healing process.

The non-healing phenotype of chronic wounds is characterized by a lack of vascularization and wound re-epithelialization [66]. The wound healing process is multistep, including inflammation, angiogenesis, and tissue regeneration of the skin. These complex processes are tightly controlled by the interplay of different cell types in the wounded tissues, including inflammatory cells, fibroblasts, keratinocytes, and endothelial cells [67,68]. IronQ-treated PBMCs significantly enhance angiogenesis (tube formation) by secreting growth factors and cytokines. IronQ-preconditioning of PBMCs may result in more favorable vascular regeneration condition or tissue repair, because of the orchestration of the dynamic expression of multiple cytokines and growth factors. The latter effects were presumably the result of IronQ-preconditioning of PBMCs accelerating the phenotypes of macrophages, T lymphocytes, and proangiogenic cells. These secreted agents also effectively induce migration of fibroblasts and endothelial cells (HUVECs). Therefore, this efficacious outcome of IronQ-preconditioning of PBMCs provides a proof of principle for the therapeutic efficacy of IronQ-preconditioning of PBMCs.

Previous publications have strongly supported our interest in the clinical applications of IronQ-treated PBMCs to treat hard-to-heal wounds or chronic skin defects (ulcers, diabetic wounds). During a first clinical prospective phase I study using the autologous secretome of PBMCs in humans, Simader et al. showed that such a treatment was safe and well-tolerated [69]. Recently, Gugerell et al. announced success in the first clinical phase II trial using the topically administered cell-free secretome of peripheral blood mononuclear cells (APOSEC) in patients suffering from diabetic foot ulcers [70]. Other teams have demonstrated animal models that expanded PBMCs as a relevant therapeutic vector for chronic skin wound healing. Tanaka et al. have shown that ex vivo-expanded PBMCs, transplanted into euglycemic and diabetic wounds in mice, led to wound closure acceleration, maturation, and vascularization [71]. Comparative effects were observed by Mildner et al., who used the secretome obtained from PBMCs to improve the wound healing response in a murine model [72]. In pigs, the transplantation of endothelial progenitor cells (EPCs) was used to promote wound closure and angiogenesis. This work was published by Kado et al., who were able to enrich PBMCs with EPCs and transplant them into the wounds of cyclosporine-immunosuppressed pigs [73]. Therefore, these data from the literature and our results contribute to indicating that IronQ-preconditioning of PBMCs is a promising approach for treating chronic wounds.

## 4. Materials and Methods

### 4.1. Chemicals and Reagents

Roswell Park Memorial Institute (RPMI) 1640 medium and Dulbecco’s Modified Eagle’s Medium/Nutrient Mixture F-12 (DMEM/F-12) were purchased from Caisson Lab (Smithfield, UT, USA). Trypsin-EDTA, fetal bovine serum, penicillin, and streptomycin were purchased from Gibco company (Gibthai, Bangkok, Thailand). 3-(4,5-dimethylthiazol-2-yl)-2,5-diphenyltetrazolium bromide (MTT), bovine serum albumin (BSA), human insulin, epidermal growth factor, basic fibroblast growth factor, and hydrocortisone were purchased from Sigma-Aldrich (St. Louis, MO, USA). Vascular endothelial growth factor was obtained from Thermo Fisher Scientific (Rockford, IL, USA). The iron (III)–quercetin complex (IronQ) was provided by Dr. Nathupakorn Dechsupa.

### 4.2. Ethics Statement

The peripheral blood mononuclear cells (PBMCs) used in this study were obtained from healthy human peripheral blood (age 20–40 years, *n* = 8), and all donors provided informed consent. The study was performed in accordance with the 2013 WMA Declaration of Helsinki. The study design was approved by the Human Research Ethics Committee of the Faculty of Medicine, Chiang Mai University (ref. no. NONE-2560-05052).

### 4.3. Cell Isolation and Culture

The PBMCs were obtained from a buffy coat bag from healthy donor volunteers at the Blood Bank Unit (Maharaj Nakorn Chiang Mai Hospital, Faculty of Medicine, Chiang Mai University). The PBMCs were isolated from 100 mL of healthy blood donor buffy coat from both male and female donors. PBMCs were isolated over a Ficoll gradient (Lymphoprep™, Stemcell Technologies, Vancouver, BC, Canada) using the manufacture’s protocol. Mononucleated cell fractions were collected and cultured in RPMI1640 medium, with L-glutamine supplemented with 10% fetal bovine serum (FBS) and 1% penicillin/streptomycin. Human umbilical vein endothelial cells (HUVECs) were purchased from American Type Culture Collection (Manassas, VA, USA). The HUVECs were cultured in an endothelial growth medium containing media DMEM/F-12 (10% fetal bovine serum + 1% penicillin/streptomycin) supplement mix, containing epidermal growth factor 5 ng/mL, basic fibroblast growth factor 10 ng/mL, insulin-like growth factor 20 ng/mL, vascular endothelial growth factor 0.5 ng/mL, heparin 22.5 µg/mL, and hydrocortisone 0.2 µg/mL; it was defined as EGM medium. L929 Mouse fibroblasts were purchased from American Type Culture Collection (Manassas, VA, USA). L929 were cultured in RPMI1640 medium with L-glutamine supplemented with 10% fetal bovine serum (FBS) and 1% penicillin/streptomycin. All cells were cultured in an incubator at 37 °C in a humidified atmosphere with 5% CO_2_. 

### 4.4. Iron–Quercetin Complex (IronQ) Treatment

Fresh PBMCs were seeded on 6-well plates at a density of 2 × 10^6^ cells/well in adequate RPMI 1640 (10% FBS and 1% penicillin/streptomycin), with or without 125 ug/mL Iron–Quercetin complex, and cultured at 37 °C in a humidified atmosphere with 5% CO_2_. Experiments were performed after 7, 10, and 14 days of culturing without subculture or re-feeding. The change in cell morphology was observed using an inverted microscope (Nikon, ECLIPSE Ts2, Tokyo, Japan).

### 4.5. Cell Proliferation Assay

The effect of IronQ on cell proliferation was determined by 3-(4,5-dimethylthiazol-2-yl)-2,5-diphenyl tetrazolium bromide (MTT) assay. Cells (1 × 10^6^ cells/well) were seeded on 24-well plates in the presence of 125 µg/mL IronQ for 1, 3, 5, 7, and 10 days. At the end time point, 200 µL MTT (5 mg/mL) was added to each well, and the cells were further incubated for 4 h. Then, the cultured supernatant was removed and 500 µL of dimethyl sulfoxide (DMSO) was added. The intensity of the formazan solution was determined by measurement of the absorbance at 560 nm using a reader (BioTekTM Eon^TM^ microplate reader, Winooski, VT, USA).

### 4.6. Flow Cytometry Analysis

The adherent post-IronQ treated PBMC cells were digested with 0.25% trypsin-EDTA and collected in phosphate buffer saline (PBS). Cell suspensions were stained at 3 × 10^5^ cells in PBS containing 0.1% BSA. Each 100 µL sample of cell suspension was incubated with IgG isotype controls or with antibodies, including anti-CD34-FITC (Miltenyi Biotec, Bergisch Gladbach, Germany), anti-CD14-FITC (Miltenyi Biotec), anti-CD11b-FITC (Invitrogen, Waltham, MA, USA), anti-CD45-PE (Invitrogen), anti-CD31-FITC (Life Technologies, Carlsbad, CA, USA), anti-CD309-PE (VEGFR-2; eBioscience, San Diego, CA, USA), and anti-105-APC (eBioscience) for 30 min at 4 °C in the dark. Then, 400 µL of PBS containing 0.1% BSA was added and analyzed using a flow cytometer (Beckman Coulter, Brea, CA, USA). Flow cytometric data were analyzed by FlowJo10 software.

### 4.7. Preparation of PBMC Conditioned Medium

The PBMCs were resuspended in RPMI-1640 medium at a density of 10 × 10^6^ cells/well in a T-25 flask, and treated with 125 µg/mL IronQ for 10 days (post-IronQ PBMC-CM). The PMBCs were cultured in RPMI-1640 medium without IronQ as the control conditioned medium (control PBMC-CM). After 10 days of incubation, the cells were further incubated with serum-free RPMI-1640 medium for 24 h. The cell suspension and conditioned medium were collected and centrifuged to eliminate cells and cellular debris, and filtered by passing through a filter of 0.22 µm pore size (Millex-Millipore™, Millipore Corporation, Burlington, MA, USA).

### 4.8. Enzyme-Linked Immunosorbent Assay (ELISA)

The secreted cytokines from the PBMC conditioned medium were collected and investigated by enzyme-linked immunosorbent assay (ELISA). Conditioned medium from PBMCs cultured without IronQ (PBMC control-CM) or from PBMCs post-IronQ treatment (post-IronQ PBMC-CM) was tested for the presence of IL-8, vascular endothelial growth factor (VEGF), IL-10, monocyte chemoattractant protein-1 (MCP-1), matrix metallopeptidase 9 (MMP-9), tumor necrosis factor-alpha (TNF-α), plasminogen activator inhibitor-1 (PAI-1), and urokinase-type plasminogen activator (uPA) by human ELISA kit (Invitrogen, USA), strictly following the manufacturer’s instructions.

### 4.9. Life Cell Labeling

The adherent post-IronQ treated PBMCs were digested with 0.25% trypsin-EDTA and collected in phosphate buffer saline (PBS). According to the manufacturer’s instructions, the cells were labeled with green fluorescence (PKH67 Fluorescent cell linker kits; Sigma-Aldrich, Burlington, MA, USA).

### 4.10. Tube Formation Assay on Matrigel

To investigate the angiogenic potential of post-IronQ PBMCs, Matrigel (Geltrex TM, Life Technologies, Carlsbad, CA, USA) was used for in vitro tube formation assay. Briefly, Matrigel solution was added to 96-well plates at 37 °C for 1 h to allow the matrix solution to solidify. A total of 1 × 10^4^ cells HUVECs were seeded and cultured in 250 µL EGM medium or supernatant culture medium from pre-and-post-IronQ PBMCs. Where indicated, the post-IronQ PBMCs were harvested and labeled with PKH67 fluorescent cell linker and re-plated (5 × 10^3^ cells/well), and co-cultured with HUVECs on the solidified matrix solution. Cells seeded on Matrigel were incubated at 37 °C. The tubule-like formation was observed under an inverted light microscope. To evaluate the angiogenic capacity, the total length of the tubes formed during the assay was analyzed by Wimasis Image Analysis (Cordoba, Spain). Post-IronQ treated PBMC-labeled cells incorporated into the tube formation were observed and captured using fluorescence microscopy (Nikon, ECLIPSE Ts2, Tokyo, Japan).

### 4.11. HUVEC Migration with Conditioned Medium from Post-IronQ Treated PBMCs

HUVECs were serum-starved in EGM medium containing 2% FBS for 12 h. Then, cells (1 × 10^5^ cells) were seeded at the upper chamber of Transwell inserts of 24-well plates with an 8 mm membrane pore size (Corning Life Sciences, Tewksbury, MA, USA). The different conditioned mediums were added as a chemoattractant in the lower chamber. After 12 h, the medium was aspirated, and the non-migrated cells in the upper surface of each membrane were removed by gentle swabbing. Membranes were fixed with methanol and stained with 0.5% crystal violet solution (Sigma-Aldrich, Burlington, MA, USA). The stained cells were photographs from four random fields of view for each membrane under an inverted microscope (Nikon, ECLIPSE Ts2, Tokyo, Japan).

### 4.12. Scratch Wound-Induced Fibroblast Migration Assay

L929 fibroblast cells were seeded in 6-well plates at a density of 2 × 10^5^ cells/well. When the cells reached the whole confluence of the well, cells were serum-starved overnight by incubated in a serum-free medium a starvation medium. Cell monolayers were wounded with a sterile 200 µL pipette tip, and washed with phosphate-buffered saline (PBS) to remove the detached cells from the plates. The cells were left untreated or treated with a conditioned medium, and kept at 37 °C in a CO_2_ incubator for 24 h. The wound gap was observed and photographed using phase-contrast microscopy (Nikon, ECLIPSE Ts2, Tokyo, Japan). The images were then analyzed using Image J software 1.52v version (National Institute of Health, Bethesda, MD, USA) to measure the width of the scratch.

### 4.13. Statistical Analysis

The statistical analysis of the mean comparison was performed by using the OriginPro version 2018 program (Northampton, MA, USA) or IBM^®^ SPSS^®^ Statistics Subscription software (IBM Corp. in Armonk, NY, USA). A paired t-test was performed to examine the levels of MCP-1, VEGF, PAI-1, uPA, IL-8, IL-10, MMP-9, and TNF-α secreted into PBMC-CMs. One-way analysis of variance (ANOVA) followed by Tukey’s or Bonferroni’s post hoc tests were used to analyze (1) kinetic surface marker expression in PBMCs exposed to the IronQ, (2) HUVEC networking formation, (3) the migration of HUVECs, and (4) the migration of fibroblasts. Data are expressed as mean ± standard deviation (SD). A value of *p* < 0.05 was considered statistically significant.

## 5. Conclusions

The novel technique of IronQ-preconditioning of PBMCs investigated in this study could be an alternative method for cell expansion and therapy for ischemic diseases and chronic wounds. It has the advantage of using paramagnetic agents to expand the limited number of progenitor cells from non-mobilized PBMCs without the addition of growth factors and can serve as the magnetic label for MRI at the same time. However, further study is the warranted for a better understanding of the mechanism underlying the effect of IronQ on PBMCs, alongside in vivo assessments in animal models of non-healing wounds.

## Data Availability

Not applicable.
